# Parasitic copepods (Crustacea, Hexanauplia) on fishes from the lagoon flats of Palmyra Atoll, Central Pacific

**DOI:** 10.3897/zookeys.833.30835

**Published:** 2019-03-27

**Authors:** Lilia C. Soler-Jiménez, F. Neptalí Morales-Serna, Ma. Leopoldina Aguirre-Macedo, John P. McLaughlin, Alejandra G. Jaramillo, Jenny C. Shaw, Anna K. James, Ryan F. Hechinger, Armand M. Kuris, Kevin D. Lafferty, Victor M. Vidal-Martínez

**Affiliations:** 1 Laboratorio de Parasitología, Centro de Investigación y de Estudios Avanzados del IPN (CINVESTAV-IPN) Unidad Mérida, Carretera Antigua a Progreso Km. 6, Mérida, Yucatán C.P. 97310, México; 2 CONACYT, Centro de Investigación en Alimentación y Desarrollo, Unidad Académica Mazatlán en Acuicultura y Manejo Ambiental, Av. Sábalo Cerritos S/N, Mazatlán 82112, Sinaloa, México; 3 Department of Ecology, Evolution and Marine Biology and Marine Science Institute, University of California, Santa Barbara CA 93106, USA; 4 Scripps Institution of Oceanography-Marine Biology Research Division, University of California, San Diego, La Jolla, California 92093 USA; 5 Western Ecological Research Center, U.S. Geological Survey, Marine Science Institute, University of California, Santa Barbara CA 93106, USA

**Keywords:** Parasitic copepods, fish, geographical isolation, islands, Indo-Pacific, atoll

## Abstract

We surveyed copepods parasitic on the fishes at Palmyra, a remote atoll in the Central Indo-Pacific faunal region. In total, we collected 849 individual fish, representing 44 species, from the intertidal lagoon flats at Palmyra and recovered 17 parasitic copepod species. The parasitic copepods were: *Orbitacolaxwilliamsi* on *Mulloidichthysflavolineatus*; *Anuretesserratus* on *Acanthurusxanthopterus*; *Caligusconfusus* on *Carangoidesferdau*, *Carangoidesorthogrammus*, *Caranxignobilis*, *Caranxmelampygus*, and *Caranxpapuensis*; *Caliguskapuhili* on *Chaetodonauriga* and *Chaetodonlunula*; *Caliguslaticaudus* on *Rhinecanthusaculeatus*, *Pseudobalistesflavimarginatus*, *M.flavolineatus*, *Upeneustaeniopterus*, *Chrysipteraglauca*, and *Epinephalusmerra*; *Caligusmutabilis* on *Lutjanusfulvus* and *Lutjanusmonostigma*; *Caligusrandalli* on *C.ignobilis*; *Caligus* sp. on *L.fulvus*; *Caritusserratus* on *Chanoschanos*; *Lepeophtheiruslewisi* on *A.xanthopterus*; *Lepeophtheirusuluus* on *C.ignobilis*; *Dissonussimilis* on *Arothronhispidus*; *Nemesis* sp. on *Carcharhinusmelanopterus*; *Hatschekialongiabdominalis* on *A.hispidus*; *Hatschekiabicaudata* on *Chaetodonauriga* and *Chaetodonlunula*; *Kroyerialongicauda* on *C.melanopterus* and *Lernanthropus* sp. on *Kyphosuscinerascens*. All copepod species reported here have been previously reported from the Indo-Pacific but represent new geographical records for Palmyra, demonstrating large-scale parasite dispersion strategies.

## Introduction

Although there have been several surveys of copepods parasitic on Indo-Pacific fishes, including the Great Barrier Reef (Australia), New Caledonia, New Guinea, India, Taiwan and the Hawaiian Islands (Yamaguti 1963, Kabata 1966, Lewis 1968, Pillai 1968, 1985, Cressey and Boyle 1973, Cressey and Cressey 1979, Ho and Dojiri 1977, Deets and Dojiri 1990, Ogawa 1991, Ho and Lin 2004, Boxshall and Justine 2005, Tang and Kalman 2005, Palm and Bray 2014), the East Indo-Pacific has received little sampling effort. Lafferty et al. (2008) compared parasite communities, including parasitic copepods, at two coral atolls in the Line Islands chain of the central Pacific (Kiritimati Island and Palmyra Atoll). However, their analysis was limited to broad patterns of richness and abundance of morphospecies, conservatively grouped into broad taxonomic categories. Palm and Bray (2014) listed parasites from Hawaiian fishes, reporting 64 copepod species (13 families) from 298 identified fish species.

Palmyra Atoll is one of the northern Line Islands located in the Indo-Pacific (IP) marine ecoregion (Spalding et al. 2007), 1680 km SSW of Hawaii. It is presently a marine protected area and has not supported regular human settlement since World War II. Palmyra Atoll has a relatively long history with little to no exploitation (DeMartini et al. 2008, Sandin et al. 2008). All fishing has been prohibited at Palmyra since it became a US National Wildlife Refuge in 2000 (before that, its remoteness kept fishing pressure low).

As part of a larger project assembling food webs at Palmyra Atoll, we have been cataloging the parasites found in the system. This paper is a companion to two others examining different fish parasite taxa (Vidal-Martínez et al. 2012, 2017). We recovered a considerable number of parasitic copepods from 44 fish species. As such, our tabulation adds to the few published species descriptions or host records from the Central Indo-Pacific region (Cressey and Boyle 1973, 1979, Cressey 1977, Ho and Lin 2004, Palm and Bray 2014), with an emphasis on describing diversity of the copepod supracommunity (Bush et al. 1997) at this site. The goal of this study is two-fold. First, we list the copepod species recovered, and note, for each, taxonomic issues and report their prevalence, mean intensity and host species. Second, we then discuss this diversity survey with respect to previously published records for the region.

## Material and methods

We collected fish by seine, spear, and hook and line from the intertidal sand flats bordering the lagoon of Palmyra Atoll between October 2009 and July 2012. To avoid loss or mixing of parasites among fishes, immediately after capture, we placed fish in individual plastic bags with lagoon water and transported them to the laboratory facility of the Palmyra Atoll Research Consortium (PARC). We examined only freshly killed fish (and the bag water). Observations were under a stereomicroscope. Skin and fins of each host were carefully examined. The gill arches were removed and examined under a stereomicroscope. The copepods obtained were counted, preliminarily identified, fixed in 95 % EtOH, labelled and stored in vials for later evaluation. Then, in the Laboratory of Aquatic Pathology of CINVESTAV-Mérida, specimens were mounted and cleared with lactophenol to identify species based on morphology using an Olympus BX-53 microscope (Olympus Corporation, Shinjuku, Tokyo, Japan). Prevalence and mean intensity concepts were applied following Bush et al. (1997). Synonyms for each host species and copepod species were obtained from FishBase (Froese and Pauly 2018) and World of Copepods (Walter and Boxshall 2018), respectively. Voucher specimens were deposited in the United States National Parasite Collection, Washington, DC (USNPC), and the Helminthological Collection of the Laboratory of Parasitology, at the Centre for Research and Advanced Studies, National Polytechnic Institute, Mérida, Yucatán, México (CHCM).

## Results

### Copepods of fishes from Palmyra lagoon flats

During this study, 849 individual fish from 44 species were collected. Fourteen of the 44 fish species examined were parasitized by at least one parasitic copepod species. *Caranxignobilis* (Forsskål) was host to three copepod species, the most of any fish. *Acanthurusxanthopterus* Valenciennes, *Carcharhinusmelanopterus* (Quoy & Gaimard), *Chaetodonauriga* Forsskål, *Chaetodonlunula* (Lacépède), *Lutjanusfulvus* (Forster), *Mulloidichthysflavolineatus* (Lacépède) and *Arothronhispidus* (Linnaeus) served as host for two copepod species. All other infected species hosted a single copepod species. Thirty fish species were found free of any copepod parasite (Table 1). Ten of the 17 copepod species recovered belong to the Caligidae family (Table 2).

**Table 1.** Fish species examined from the lagoon flats from the Palmyra Atoll. N = number of fish examined; Max = maximum length reported for that fish species in FishBase (http://www.fishbase.se); Range = total length range of the fish examined.

Host examined	Fish common name	N	Infected hosts	Max (cm)	Range (cm)
** Acanthuridae **					
*Acanthurustriostegus* (Linnaeus, 1758)	Convict surgeonfish	50	0	27	10–18
*Acanthurusxanthopterus* Valenciennes, 1835	Yellowfin surgeonfish	20	2	70	20–40
** Albulidae **					
*Albulaglossodonta* (Forsskål, 1775)	Roundjaw bonefish	24	0	90	37–58
** Apogonidae **					
*Cheilodipterusquinquelineatus* Cuvier, 1828	Five-lined cardinalfish	5	0	13	5–6
** Balistidae **					
*Pseudobalistesflavimarginatus* (Rüppell, 1829)	Yellowmargin triggerfish	4	0	60	17–53
*Rhinecanthusaculeatus* (Linnaeus, 1758)	Blackbar triggerfish	18	0	30	8–24
** Belonidae **					
*Platybeloneargalus* (Lesueur, 1821)	Keeltail needlefish	2	0	50	9–36
** Carangidae **					
*Carangoidesferdau* (Forsskål, 1775)	Blue trevally	5	0	75	33–38
*Carangoidesorthogrammus* (Jordan & Gilbert, 1882)	Island trevally	3	0	75	25–35
*Caranxignobilis* (Forsskål, 1775)	Giant trevally	4	3	170	56–79
*Caranxmelampygus* Cuvier, 1833	Bluefin trevally	6	2	117	31–66
*Caranxpapuensis* Alleyne & MacLeay, 1877	Brassy trevally	5	2	88	12–41
** Carcharhinidae **					
*Carcharhinusmelanopterus* (Quoy & Gaimard, 1824)	Blacktip reef shark	5	3	200	46–219
** Chaetodontidae **					
*Chaetodonauriga* Forsskål, 1775	Threadfin butterflyfish	13	4	23	12–19
*Chaetodonlunula* (Lacepède, 1802)	Raccoon butterflyfish	14	6	20	11–16
** Chanidae **					
*Chanoschanos* (Forsskål, 1775)	Milkfish	5	1	180	31–57
** Gobiidae **					
*Amblygobiusphalaena* (Valenciennes, 1837)	Whitebarred goby	18	0	15	1.3–7
*Asterropteryxsemipunctata* Rüppell, 1830	Starry goby	12	0	6	2–4
*Gnatholepisanjerensis* (Bleeker, 1851)	Eye-bar goby	2	0	8	2–3
*Istigobiusdecoratus* (Herre, 1927)	Decorated goby	5	0	13	7–11
*Istigobiusornatus* (Rüppell, 1830)	Ornate goby	26	0	11	3–6
*Istigobiusrigilius* (Herre, 1953)	Rigilius goby	1	0	11	4
*Oplopomusoplopomus* (Valenciennes, 1837)	Spinecheek goby	26	0	10	2–7
*Psilogobiusprolatus* Watson & Lachner, 1985	Longjaw shrimpgoby	11	0	6	2–4
*Valencienneasexguttata* (Valenciennes, 1837)	Sixspot goby	14	0	14	2–9
** Hemiramphidae **					
*Hemiramphusdepauperatus* Lay & Bennett, 1839	Tropical half-beak fish	20	0	40	20–34
** Kiphosidae **					
*Kyphosuscinerascens* (Forsskål, 1775)	Blue sea chub	2	1	50	35–38
** Lutjanidae **					
*Lutjanusfulvus* (Forster, 1801)	Blacktail snapper	26	5	40	7–26
*Lutjanusmonostigma* (Cuvier, 1828)	One spot snapper	6	1	60	17–37
** Mugilidae **					
*Crenimugilcrenilabis* (Forsskål, 1775)	Fringelip mullet	42	0	60	8–45
*Lizavaigiensis* (Quoy & Gaimard, 1825)	Squaretail mullet	54	0	63	3–32
*Valamugilengeli* (Bleeker, 1858)	Kanda	63	0	30	1–20
** Mullidae **					
*Mulloidichthysflavolineatus* (Lacepède, 1801)	Yellowstripe goatfish	52	8	43	8–37
*Upeneustaeniopterus* Cuvier, 1829	Finstripe goatfish	5	3	33	1–30
** Muraenidae **					
*Gymnothoraxpictus* (Ahl, 1789)	Paintspotted moray	7	0	140	41–70
** Ophichthidae **					
*Myrichthyscolubrinus* (Boddaert, 1781)	Harlequin snake eel	3	0	97	33–65
** Pinguipedidae **					
*Parapercislata* Randall & McCosker, 2002	Y-Barred Sandperch	13	0	21	2–3
** Pomacentridae **					
*Abudefdufseptemfasciatus* (Cuvier, 1830)	Banded sergeant	12	0	23	14–20
*Abudefdufsordidus* (Forsskål, 1775)	Blackspot sergeant	18	0	24	14–19
*Chrysipteraglauca* (Cuvier, 1830)	Grey demoiselle	3	0	12	8–10
*Stegastesnigricans* (Lacepède, 1802)	Dusky farmerfish	10	0	14	8–10
** Serranidae **					
*Epinephelusmerra* Bloch, 1793	Honeycomb grouper	2	0	32	13–24
** Sphyraenidae **					
*Sphyraenabarracuda* (Edwards, 1771)	Great barracuda	2	0	200	65–76
** Tetraodontidae **					
*Arothronhispidus* (Linnaeus, 1758)	White-spotted puffer	15	9	50	17–49

**Table 2.** Parasitic copepods of fishes from the lagoon flats of Palmyra Atoll; N = number of fish examined. The authorities for parasites were included in the text.

**Copepod species**	**Hosts**	**N**	**Infected hosts**	**Prevalence (%)**	**Mean intensity (± SD)**
** Bomolochidae **					
* Orbitacolax williamsi *	* Mulloidichthys flavolineatus *	52	1	1.9	1
** Caligidae **					
* Anuretes serratus *	* Acanthurus xanthopterus *	20	1	5	6
* Caligus confusus *	* Carangoides ferdau *	5	2	40	2 ± 0.0
	* Carangoides orthogrammus *	3	1	33.3	6
	* Caranx ignobilis *	4	3	75	12.7 ± 12.2
	* Caranx melampygus *	6	2	40	4 ± 0.0
	* Caranx papuensis *	5	2	33.3	2 ± 0.0
* Caligus kapuhili *	* Chaetodon auriga *	13	1	7.7	8
	* Chaetodon lunula *	14	4	28.6	2.5 ± 1.7
* Caligus laticaudus *	* Rhinecanthus aculeatus *	18		5.6	1
	* Pseudobalistes flavimarginatus *	4	2	50	21 ± 26.9
	* Mulloidichthys flavolineatus *	52	7	13.5	1.5 ± 0.5
	* Upeneus taeniopterus *	5	3	60	2.7 ± 2.1
	* Chrysiptera glauca *	3	1	3.33	2
	* Epinephalus merra *	2	1	50	1
* Caligus aff. mutabilis *	* Lutjanus fulvus *	26	4	15.4	1.75 ± 1.5
	* Lutjanus monostigma *	6	1	16.6	2
* Caligus randalli *	* Caranx ignobilis *	4	1	25	1
*Caligus* sp.	* Lutjanus fulvus *	26	1	3.8	1
* Caritus serratus *	* Chanos chanos *	5	1	20	4
* Lepeophtheirus lewisi *	* Acanthurus xanthopterus *	20	1	5	1
* Lepeophtheirus uluus *	* Caranx ignobilis *	4	1	25	4
** Dissonidae **					
* Dissonus similis *	* Arothron hispidus *	15	2	13.3	2 ± 0.0
** Eudactylinidae **					
*Nemesis* sp.	* Carcharhinus melanopterus *	5	2	40	2 ± 0.0
** Hatschekiidae **					
* Hatschekia longiabdominalis *	* Arothron hispidus *	15	8	53.3	100 ± 329.2
* Hatschekia bicaudata *	* Chaetodon auriga *	13	3	23.1	7.3 ± 3.1
	* Chaetodon lunula *	14	2	14.3	5 ± 1.4
** Kroyeriidae **					
* Kroyeria longicauda *	* Carcharhinus melanopterus *	5	2	40	16 ± 2.8
** Lernanthropidae **					
*Lernanthropus* sp.	* Kyphosus cinerascens *	2	1	50	2

### Order Cyclopoida Milne Edwards, 1840

#### Bomolochidae Claus, 1875

##### *Orbitacolax* Shen, 1957

###### Orbitacolax williamsi

Taxon classificationAnimaliaCyclopoidaBomolochidae

Cressey & Cressey, 1989

####### Type host.

*Scolopsistaenioptera* (as *S.dubiosus*) (Cuvier) (Nemipteridae).

####### Other host and localities.

*Scolopsistaenioptera* (as *S.dubiosus*) from Okinawa, Japan (Cressey and Cressey 1989). *Corisbatuensis* (Bleeker) (Labridae) from Lizard Island, Australia (Muñoz and Cribb 2006). *Thamnaconusdegeni* (Regan) (Monacanthidae) from South Australia (Hayward et al. 2011).

####### Current host.

*Mulloidichthysflavolineatus* (Mullidae).

####### Site of infection.

Gills.

####### Prevalence and mean intensity.

1.9 and 1 (n = 52).

####### Specimens deposited.

CHCM No. 560 (voucher) (1 vial, 1 specimen ♀).

####### Remarks.

To date, the genus *Orbitacolax* includes 10 valid species, which form two clusters (Venmathi Maran et al. 2014), the *hapalogenyos*-group with four species (*O.hapalogenyos*, *O.pteragogi*, *O.trichiuri*, and *O.unguifer*) and *analogus*-group with six species (*O.analogus*, *O.dactylopterusi*, *O.aculeatus*, *O.leptoscari*, *O.uniunquis*, and *O.williamsi*). This second group is based on the second endopodal segment of leg 2 either no inner seta or having 1 inner seta. Particularly, *O.williamsi* lacks seta on the second endopodal segment of leg 2, as seen in our specimen and the original description provided by Cressey and Cressey (1989). However, Venmathi-Maran et al. (2014) pointed out that *O.williamsi* carries 1 inner seta in that segment, but this is likely inaccurate. *Orbitacolaxwilliamsi* has been found on western Pacific fishes from four families, suggesting that this parasite may have a low host specificity.

### Order Siphonostomatoida Burmeister, 1835

#### Caligidae Burmeister, 1834

##### *Anuretes* Heller, 1865

###### Anuretes serratus

Taxon classificationAnimaliaSiphonostomatoidaCaligidae

Shiino, 1954

####### Type host.

*Prionurusscalprum* (as *Xesurusscalprum*) Valenciennes (Acanthuridae).

####### Other host and localities.

*Prionurusscalprum* (as *Xesurusscalprum*) (Acanthuridae) from Seto, Wakayama Prefecture, Japan (Shiino 1954). *Nasohexacanthus* (Bleeker) (Acanthuridae) from Oahu, Hawaii (Lewis 1964a, Palm and Bray 2014); from Japan and India (Prabha and Pillai 1986). *Prionurusmicrolepidotus* Lacepède (Acanthuridae) from Australia (Boxshall 2018).

####### Current host.

*Acanthurusxanthopterus* (Acanthuridae).

####### Site of infection.

Gills.

####### Prevalence and mean intensity.

5 and 6 (n = 20).

####### Specimens deposited.

CHCM No. 561 (voucher) (1 vial, 1 specimen ♀).

####### Remarks.

The validity of the genus *Anuretes* is questionable given the considerable morphological overlap with the members of *Lepeophtheirus* (Dojiri and Ho 2013). Currently, *Anuretes* includes 21 valid species (Boxshall 2018, Walter and Boxshall 2018); of which *A.serratus* may be distinguished by stout spines on distal exopodal segment of leg 1, and a branched spine on first exopodal segment of leg 2 (Shiino 1954, Lewis 1964a), which were clearly observed in our specimens. In addition, *A.serratus* lacks sternal furca. According to Dojiri and Ho (2013), a sternal furca is rarely absent in species of *Anuretes*.

##### *Caligus* Müller, 1785

###### Caligus confusus

Taxon classificationAnimaliaSiphonostomatoidaCaligidae

Pillai, 1961

####### Type host.

*Caranxignobilis* (as *C.sansun*) (Carangidae).

####### Other host and localities.

*Alepesdjedaba* (Forsskål) from Durban; *Caranxcaballus* (Günther) and *Caranxcaninus* (Günther) from Mexican Pacific and Ecuador; *Caranxdjedaba* (Forsskål) from Durban, South Africa and Sri Lanka; *Caranxhippos* (Linnaeus) from Galapagos Islands and Panama; *Caranxignobilis* from Taiwan, Indian and Australia; *Caranxmelampygus* Cuvier from Eniwetok Atoll and Taiwan; *Caranxsexfasciatus* Quoy & Gaimard from South Africa, Taiwan, Indonesia and Australia; *Caranx* sp. from Celebes and New Caledonia (all Carangidae); *Coryphaenahippurus* Linnaeus (Coryphaenidae) from Galapagos Islands and Panama; *Decapterus* sp. (Carangidae) from Tonkin Gulf, Vietnam; *Elagatisbipinnulata* (Quoy & Gaimard) (Carangidae) from Galapagos Islands, Panama, India and Taiwan; *Elagatis* sp. from Celebes; *Epinephelustauvina* (Forsskål) (Serranidae) from Kuwait; *Rhabdosargusholubi* (Steindachner) (Sparidae) from South Africa; *Serioladumerili* (Risso) (Carangidae) from Taiwan; *Seriola* sp. (Carangidae) from Colombia (Kabata 1968, Grobler et al. 2003, Ho and Lin 2004, Yuniar et al. 2007, Kazachenko et al. 2014, Morales-Serna et al. 2014, 2015, Boxshall 2018).

####### Current host.

*Carangoidesferdau* (Forsskål), *Carangoidesorthogrammus* (Jordan & Gilbert), *Caranxignobilis*, *Caranxmelampygus* and *Caranxpapuensis* Alleyne & MacLeay (all Carangidae).

####### Site of infection.

Gills.

####### Prevalence and mean intensity.

40 and 2 (n = 5) to *Carangoidesferdau*, 33.3 and 6 (n = 3) to *Carangoidesorthogrammus*, 75 and 12.7 ± 12.2 (n = 4) to *Caranxignobilis*; 33.3 and 2 (n = 6) to *Caranxmelampygus*; 40 and 4 (n = 5) to *Caranxpapuensis*.

####### Specimens deposited.

CHCM No. 562 (voucher) (1 vial, 1 specimen ♀) (from *Caranxignobilis*), CHCM No. 563 (voucher) (1 vial, 2 specimens ♂ ♀) (from *Caranxpapuensis*), USNM No. 1550598 (voucher) (1 vial, 1 specimen ♀) (from *Caranxignobilis*).

####### Remarks.

The genus *Caligus* contains approximately 250 species. According to Ho and Lin (2004), before the establishment of *C.confusus*, specimens of this species were confused with *Caligusproductus* (as *Caligusalalongae*) Dana, 1852 and *Caligusconstrictus* Heller, 1865. However, these authors pointed out nine characteristics known only for *C.confusus*. The morphology of our specimens (♂ and ♀) fits with the description of Ho and Lin (2004). Additionally, based on the examination of the present material and also that from previous surveys in the Eastern Pacific (Morales-Serna et al. 2014, 2015), we suggest that the shape of the first segment of the antenna and sternal furca may be useful in identifying *C.confusus*. Clearly, *C.confusus* has high affinity for carangid fish; nonetheless, this parasite can also be found on fish from different families. To date, it is distributed in tropical waters of the Eastern Pacific and Indo-Pacific, with no records for the Atlantic Ocean.

###### Caligus kapuhili

Taxon classificationAnimaliaSiphonostomatoidaCaligidae

Lewis, 1967

####### Type host.

*Chaetodonmiliaris* Quoy & Gaimard (Chaetodontidae).

####### Other host and localities.

*Chaetodonmiliaris* Quoy & Gaimard, *Chaetodonfremblii* Bennett from Hawaii (Lewis 1967, Palm and Bray 2014). *Chaetodonauripes* Jordan & Snyder and *Chaetodonvagabundus* Linnaeus from Taiwan (all Chaetodontidae) (Ho and Lin 2007).

####### Current host.

*Chaetodonauriga* and *Chaetodonlunula* (Chaetodontidae).

####### Site of infection.

Gills.

####### Prevalence and mean intensity.

7.7 and 8 (n = 13) to *Chaetodonauriga*; 28.6 and 2.5 ± 1.7 (n = 14) to *Chaetodonlunula*.

####### Specimens deposited.

CHCM No. 564 (voucher) (1 vial, 1 specimen ♂) (from *C.auriga*). CHCM No. 565 (voucher) (1 vial, 1 specimen ♂) (from *C.lunula*). USNM No. 1550599 (voucher) (1 vial, 1 specimen ♂) (from *C.lunula*).

####### Remarks.

According to Lewis (1967) and Lin and Ho (2007), *C.kapuhili* is morphologically close to *Caliguslaticaudus* Shiino, 1960. However, the abdomen is 1-segmented in *C.kapuhili* and 2-segmented in *C.laticaudus*. We found specimens of *C.laticaudus* (see below), which facilitated our morphological analysis. Likewise, we identified *C.kapuhili* based on host preference, since this species has only been found on fish of the genus *Chaetodon* from the North-West Pacific.

###### Caligus laticaudus

Taxon classificationAnimaliaSiphonostomatoidaCaligidae

Shiino, 1960

####### Type host.

*Pagrusmajor* (as *Pagrosomusmajor*) (Temminck & Schlegel) (Sparidae).

####### Other host and localities.

*Pagrusmajor* (as *Pagrosomusmajor*) (Sparidae) from Japan (Shiino 1960). *Acanthurusolivaceus* Bloch & Schneider (Acanthuridae) from Eniwetok Atoll; *Dentextumifrons* (Temminck & Schlegel) (Sparidae) from Korea; *Lizahaematocheila* (Temminck & Schlegel) (Mugilidae) from China; *Caranxmelampygus* (Carangidae), *Lutjanusvitta* (Quoy & Gaimard), *Lutjanusrussellii* (Bleeker) (Lutjanidae) and *Parapristipomatrilineatum* (Thunberg) (Haemulidae), *Polydactylusplebeius* (Broussonet) and *Polydactylussextarius* (Bloch & Schneider) (Polynemidae) from Taiwan; *Parastomateusniger* (Bloch) (Carangidae) from Malaysia; *Filimanusheptadactyla* (Cuvier) (Polynemidae) and *Rhabdosargussarba* (Forsskål) (Sparidae) from India (Ho and Lin 2004, Moon and Kim 2012). *Gnathanodonspeciosus* (Forsskål), *Caranxsexfasciatus* Quoy & Gaimard (Carangidae), *Heniochusacuminatus* (Linnaeus) (Chaetodontidae), *Kyphosusbigibbus* Lacepède (Kiphosidae), *Pseudolabrusguentheri* Bleeker (Labridae), *Pagrusauratus* (Forster) (Sparidae) from Australia (Boxshall 2018).

####### Current host.

*Rhinecanthusaculeatus* (Linnaeus), *Pseudobalistesflavimarginatus* (Rüppell) (Balistidae), *Mulloidichthysflavolineatus*, *Upeneustaeniopterus* Cuvier (Mullidae), *Chrysipteraglauca* (Cuvier) (Pomacentridae) and *Epinephalusmerra* Bloch (Serranidae).

####### Site of infection.

Gills..

####### Prevalence and mean intensity.

5.6 and 1 (n = 18) to *Rhinecanthusaculeatus*; 50 and 21 ± 26.9 (n = 4) to *Pseudobalistesflavimarginatus*; 13.5 and 1.5 ± 0.5 (n = 52) to *Mulloidichthysflavolineatus*; 60 and 2.7 ± 2.1 (n = 5) to *Upeneustaeniopterus*; 3.33 and 2 (n = 3) to *Chrysiptera*glauca; 50 and 1 (n = 2) to *Epinephalusmerra*.

####### Specimens deposited.

CHCM No. 566 (voucher) (1 vial, 2 specimens ♂ ♀) (from *M.flavolineatus*). USNM No. 1550600 (voucher) (1 vial, 1 specimen ♂) (from *M.flavolineatus*).

####### Remarks.

Ho and Lin (2004) indicated that the female of *C.laticaudus* may be identified by a combination of five characteristics (the corpus of the maxilliped with a large, conical protrusion in the myxal region; the terminal elements on last segment of exopod of leg 1 lack accessory processes; outermost element 1 of the four terminal elements of leg 1 exopod about one third of the length of other three elements which are subequal in length; formula of the 3-segmented exopod of leg 4 as I-0; I-0; III; and the terminal three spines on leg 4 subequal in length). Our results support the view that *C.laticaudus* infects fishes only from the Indo-West Pacific.

###### Caligus aff. mutabilis

Taxon classificationAnimaliaSiphonostomatoidaCaligidae

Wilson, 1905

####### Type host.

*Centropristisstriata* (as *Centropristesstriatus*) (Linnaeus) (Serranidae).

####### Other host and localities.

*Centropristisstriata* (as *Centropristesstriatus*) (Serranidae) from North American waters (Wilson 1905). *Acanthocybium* sp., *Euthynnus* sp., *Sarda* sp., *Scomberomorus* sp., and *Thunnus* sp. (all Scombridae) from Colombia; *Archosargusrhomboidalis* (Linnaeus) (Sparidae), *Chaetodipterusfaber* (Broussonet) (Ephippidae), *Mycteropercamicrolepis* (Goode & Bean), *Scomberomorusbrasiliensis* Collette, Russo & Zavala-Camin, *Scomberomorusmaculatus* (Mitchill) (Scombridae) and *Trachinotusgoodei* Jordan & Evermann (Carangidae) from Brazil; *Balistes* sp. (Balistidae), *Calamusbrachysomus* (Lockington) (Sparidae), *Centropomus* sp. (Centropomidae), *Chaetodipteruszonatus* (Girard) (Ephippidae), *Epinepheluslabriformis* (Jenyns) (Serranidae), *Hoplopagrusguentherii* Gill (Lutjanidae), *Katsuwonuspelamis* (Linnaeus) (Scombridae), *Kyphosuselegans* (Peters) (Kyphosidae), *Lutjanusguttatus* (Steindachner), *Lutjanusperu* (Nichols & Murphy) (Lutjanidae), *Menticirrhusundulatus* (Girard) (Sciaenidae), *Microlepidotusbrevipinnis* (Steindachner) (Haemulidae), *Mugilcephalus* (Linnaeus) (Mugilidae), *Paralabraxclathratus* (Girard), *Paralabraxmaculatofasciatus* (Steindachner), *Paralabraxnebulifer* (Girard) (all Serranidae), *Sardachiliensis* (Cuvier), *Scomberomorussierra* Jordan & Starks (Scombridae) and *Seleneorstedii* Lütken (Carangidae) from Mexican Pacific; *S.brasiliensis* from Costa Rica; *Scomberomoruscavalla* (Cuvier) (Scombridae) from Surinam; *S.maculatus* from Florida; *Scomberomorusjaponicus* from Campeche (Gulf of Mexico); *E.labriformis*, *Eucinostomusentomelas* Zahuranec (Gerreidae), *Haemulopsisaxillaris* (Steindachner) (Haemulidae), *Paralabraxcallaensis* Starks (Serranidae), *Chromiscyanea* (Poey) and *Chromismultilineata* (Guichenot) (Pomacentridae) from Ecuador (Cressey and Cressey 1980, Luque and Tavares 2007, Gomes-Sanches et al. 2012, Morales-Serna et al. 2016).

####### Current hosts.

*Lutjanusfulvus* and *Lutjanusmonostigma* (Cuvier) (Lutjanidae).

####### Site of infection.

Gills..

####### Prevalence and mean intensity.

15.4 and 1.75 ± 1.5 (n = 26) to *L.fulvus*; 16.6 and 2 (n = 6) to *L.monostigma*.

####### Specimens deposited.

CHCM No. 567 (voucher) (1 vial, 1 specimen ♂) (from *L.fulvus*), CHCM No. 568 (voucher) (1 vial, 1 specimen ♂) (from *L.monostigma*). USNM No. 1550601 (voucher) (1 vial, 1 specimen ♂) (from *L.monostigma*).

####### Remarks.

Wilson (1905) observed that the genital complex of *C.mutabilis* varies according to the age of the individuals as well as the developmental stage of the eggs. Also, this author described *C.mutabilis* as having a short, 2-segmented abdomen. Later, Cressey and Cressey (1980) redescribed this species based on material collected from scombrid fish. These authors noted an incomplete 2-segmented abdomen and at least two other differences from the type specimens; however, such differences were not considered sufficient to propose a new species. Recently, Morales-Serna et al. (2014, 2015) reported *C.mutabilis* from different host species in the Eastern Pacific, but a molecular analysis revealed relatively high intraspecific genetic divergence among the *C.mutabilis* isolates. Our specimens share the morphological characteristics described by Cressey and Cressey (1980).

###### Caligus randalli

Taxon classificationAnimaliaSiphonostomatoidaCaligidae

Lewis, 1964

####### Type host.

*Acanthurustriostegus* (Linnaeus) (Acanthuridae).

####### Other host and localities.

To our knowledge, *C.randalli* has not been recorded since its original description (Lewis 1964a). *Acanthurustriostegus* (Acanthuridae) from Hawaii (Lewis 1964a, Palm and Bray 2014).

####### Current host.

*Caranxignobilis* (Carangidae).

####### Site of infection.

Gills.

####### Prevalence and mean intensity.

25 and 1 (n = 4).

####### Specimens deposited.

CHCM No. 569 (voucher) (1 vial, 2 specimens ♂♀). USNM No. 1550602 (voucher) (1 vial, 1 specimen ♂).

####### Remarks.

Lewis (1964a) observed that *Caligusrandalli* is morphologically close to *C.constrictus* Heller, 1865. According to this author, one of the main differences between both species is the length of the urosome. The urosome of *C.randalli* is one and a half times the length of the urosome of *C.constrictus*. In the present study, we noted that *C.randalli* resembles *Caligusaesopus* Wilson, 1921. However, the urosome in *C.aesopus* is shorter than in *C.randalli*. Hayes et al. (2012) included *C.aesopus* and another nine species of *Caligus* (*C.chorinemy* Kroyer, 1863, *C.tenax* Heller, 1865, *C.spinosurculus* Pearse, 1951, *C.germoi* Pearse, 1951, *C.rectus* Pearse, 1952, *C.confusus*, *C.cordyla* Pillai, 1963, *C.zylanica* Hameed & Pillai, 1986 and *C.equulae* Ho & Lin, 2003) within a cluster of caligid species sharing the following characteristics in the female: bifid postantennal process; bifid posterior process on the maxillule; heavily ornamented apron of the third leg; an inner rosette of large spinules and prominent rib-like structure with a bifid apex, arising near the border with the intercoxal sclerite of leg 3: a massive and strongly incurved spine on the first exopodal segment of leg 3; and a 3-segmented exopod on leg 4 armed with I,I,III spines. *Caligusrandalli* also shares these characteristics, and after a detailed examination. We confirmed that the morphological characteristic of our specimens fit with the description Lewis (1964a) for *C.randalli*. This is also supported by records of *C.randalli* in the Central Pacific.

###### Caligus

Taxon classificationAnimaliaSiphonostomatoidaCaligidae

sp.

####### Current host.

*Lutjanusfulvus* (Lutjanidae).

####### Site of infection.

Gills.

####### Prevalence and mean intensity.

3.8 and 1 (n = 26).

####### Specimens deposited.

CHCM No. 570 (voucher) (1 vial, 1 specimen ♂).

####### Remarks.

*Caligus* sp. is morphologically close to *Caliguslaticaudus*, mainly by the shape and armature of cephalothoracic appendages and legs. However, our specimen differs from *C.laticaudus* in the shape and size of the urosome. Unfortunately, the single specimen of *Caligus* sp. in our collection is not sufficient for a more detailed taxonomic study.

##### *Caritus* Cressey, 1967

###### Caritus serratus

Taxon classificationAnimaliaSiphonostomatoidaCaligidae

Cressey, 1967

####### Type host:.

*Chanoschanos* (Forsskål) (Chanidae).

####### Other host and localities.

*Chanoschanos* (Chanidae) from Nosy Bé, Madagascar (Cressey 1967). Reported as *Caritustolii* from *Tenualosatoli* (as *Hilsatoli*) (Valenciennes) (Clupeidae) from Sassoon Docks, Bombay (Rangnekar 1984).

####### Current host.

*Chanoschanos* (Chanidae).

####### Site of infection.

Gills.

####### Prevalence and mean intensity.

20 and 4 (n = 5).

####### Specimens deposited.

CHCM No. 571 (voucher) (1 vial, 1 specimen ♀).

####### Remarks.

Currently, *C.serratus* is the unique valid species included in the genus *Caritus*. Morphological characteristics of our specimens agree well with the redescription provided by Dojiri and Ho (2013).

##### *Lepeophtheirus* von Nordmann, 1832

###### Lepeophtheirus lewisi

Taxon classificationAnimaliaSiphonostomatoidaCaligidae

Hewitt, 1971

####### Type host.

*Acanthurusolivaceus* (Acanthuridae).

####### Other host and localities.

*Acanthurusolivaceus* (Acanthuridae) from Hawaii (Hewitt 1971). *Nasohexacanthus* (Bleeker), *Acanthurustriostegus* (Acanthuridae), *Myripristis* sp., *Fistulariapetimba* Lacepède (Fistulariidae) (Lewis 1964a, 1964b, Palm and Bray 2014).

####### Current host.

*Acanthurusxanthopterus* (Acanthuridae).

####### Site of infection.

Gills.

####### Prevalence and mean intensity.

5 and 1 (n = 20).

####### Specimens deposited.

CHCM No. 572 (voucher) (1 vial, 1 specimen ♂). USNM

No. 1550603 (voucher) (1 vial, 1 specimen ♂).

####### Remarks.

*Lepeophtheiruslewisi* was originally described as *Dentigrypsbifurcatus* by Lewis (1964a). However, Hewitt (1971) stated that there is not a useful character to separate *Dentigryps* Wilson, 1913 from *Lepeophtheirus* and, therefore, reassigned species of *Dentigryps* to *Lepeophtheirus*. As the name *L.bifurcatus* was preoccupied by *L.bifurcatus*Wilson 1905, Hewitt (1971) renamed Lewis’ species as *L.lewisi*. The material of the present study corresponds to a male of *L.lewisi*. The identification of this species was difficult without female specimens; nonetheless, the morphology of our material fits the description provided by Lewis (1964a) for the male of *L.lewisi*. In addition, this copepod has been mainly found in acanthurid fish from the Central Pacific as in the present work.

###### Lepeophtheirus uluus

Taxon classificationAnimaliaSiphonostomatoidaCaligidae

Lewis, 1964

####### Type host.

*Caranxmelampygus* (Carangidae).

####### Other host and localities.

*Caranxmelampygus* (Carangidae) from Oahu, Hawaii (Lewis 1964b, Palm and Bray 2014). Reported as *Dentigrypsulua* on *Caranxignobilis* from Heron Island, Australia (Ho and Dojiri 1977).

####### Current host.

*Caranxignobilis* (Carangidae).

####### Site of infection.

Gills.

####### Prevalence and mean intensity.

25 and 4 (n = 4).

####### Specimens deposited.

CHCM No. 573 (voucher) (1 vial, 2 specimens ♂♀).

####### Remarks.

*Lepeophtheirusuluus* was originally described as *Dentigrypsulua* by Lewis (1964b) and then transferred to *Lepeophtheirus* by Hewitt (1971). The morphology of our specimens corresponds to the original description.

#### Dissonidae Kurtz, 1924

##### *Dissonus* Wilson, 1906

###### Dissonus similis

Taxon classificationAnimaliaSiphonostomatoidaDissonidae

Kabata, 1966

####### Type host.

*Tetractenoshamiltoni* (Richardson) (as *Spheroideshamiltoni*) (Tetraodontidae).

####### Other host and localities.

*Tetractenoshamiltoni* (as *Spheroideshamiltoni*) (Tetraodontidae) from Queensland, Australia (Kabata 1966). *Arothronhispidus* from Philippines; *Arothronmeleagris* (Anonymous) from Guam; *Arothronnigropunctatus* (Bloch & Schneider) from Australia, Philippines and New Guinea; and *Arothronstellatus* (Anonymous) (all Tetraodontidae) from New Guinea (Tang and Kalman 2005).

####### Current host.

*Arothronhispidus* (Tetraodontidae).

####### Site of infection.

Gills.

####### Prevalence and mean intensity.

13.3 and 2 ± 0.5 (n = 15).

####### Specimens deposited.

CHCM No. 574 (voucher) (1 vial, 1 specimen ♀). USNM

No. 1550604 (voucher) (1 vial, 1 specimen ♀).

####### Remarks.

The family Dissonidae comprises only two genera, *Innaprokofevnas* Kazatchenko, 2001 with a single species (*I.orientcolae* Kazatchenko, 2001) and *Dissonus* with 12 species (*D.excavatus* Boxshall, Lin, Ho, Ohtsuka, Venmathi Maran & Justine, 2008; D.furcatus Kirtisinghe, 1950; *D.glaber* Kurtz, 1950; *D.heronensis* Kabata, 1966; *D.hoi* Tang & Kalman, 2005; *D.inaequalis* Boxshall, Lin, Ho, Ohtsuka, Venmathi Maran & Justine, 2008; *D.kapuri* (Ummerkutty, 1976); *D.manteri* Kabata, 1966; *D.nudiventris* Kabata, 1965; *D.ruvetti* Nuñes-Ruivo & Fourmanoir, 1956; *D.similis*; and *D.spinifer* Wilson, 1906).

According to Kabata (1966), *D.similis* is morphologically closer to *D.furcatus*. However, *D.similis* may be separated from *D.furcatus* and other congeners by the lack of a sternal furca or stylet and the presence of a genital spinulation extending over the anterior half to two thirds of ventral surface of genital complex (Tang and Kalman 2005, Boxshall et al. 2008). As indicated by Tang and Kalman (2005), *D.similis* is restricted to the tropical western Pacific and is highly host specific to tetraodontid fishes.

#### Eudactylinidae Wilson C.B., 1932

##### Nemesis

Taxon classificationAnimaliaSiphonostomatoidaEudactylinidae

sp. Risso, 1826

###### Current host.

*Carcharhinusmelanopterus* (Carcharhinidae).

###### Site of infection.

Gills.

###### Prevalence and mean intensity.

40 and 2 ± 0.1 (n = 5).

###### Specimens deposited.

CHCM No. 575 (voucher) (1 vial, 1 specimen ♀).

###### Remarks.

*Nemesis* is one of 12 genera in the family Eudactylinidae and includes about nine species (Mangena et al. 2014). *Nemesis* species can be divided into two groups by the relative width of the cephalothorax, free thoracic segments and genital segments (Dippenaar et al. 2008). One group (consisting of most of the species) has a fourth free thoracic segment that is much narrower than the preceding three, whereas the other (consisting of *N.lamna* only) has all four segments of about the same width (Kabata 1979). The identification and comparison of *Nemesis* species belonging to the first group is difficult because of morphological variation among individuals and the inconsistencies in the literature (Hewitt 1969, Kabata 1979).

#### Hatschekiidae Kabata, 1979

##### *Hatschekia* Poche, 1902

###### Hatschekia longiabdominalis

Taxon classificationAnimaliaSiphonostomatoidaHatschekiidae

Uyeno & Nagasawa, 2013

####### Type host.

*Arothronhispidus* (Tetraodontidae).

####### Other host and localities.

*Arothronhispidus* (Tetraodontidae) from Japan (Uyeno and Nagasawa 2013). To date, *H.longiabdominalis* has not been recorded from others host and locality.

####### Current host.

*Arothronhispidus* (Tetraodontidae).

####### Site of infection.

Gills.

####### Prevalence and mean intensity.

53.3 and 100 ± 329.2 (n = 15).

####### Specimens deposited.

CHCM No. 576 (voucher) (1 vial, 1 specimen ♀). USNM

No. 1550605 (voucher) (1 vial, 1 specimen ♀).

####### Remarks.

Of the nine genera included in the Hatschekiidae, the most speciose genus is *Hatschekia*, with approximately 140 valid species so far. According to Uyeno and Nagasawa (2013), *H.longiabdominalis* may be separated from other congeners by having a fusiform trunk with posterior lobes, the urosome markedly projecting beyond posterior lobes of the trunk, and unique intercoxal sclerites of legs 1 and 2, which strongly project from the middle of the anterior margin and bear four blunt processes on the posterior margin. We observed all of these characters in our specimens.

###### Hatschekia bicaudata

Taxon classificationAnimaliaSiphonostomatoidaHatschekiidae

Kabata, 1991

####### Type host.

*Chaetodonaureofasciatus* Macleay (Chaetodontidae).

####### Other host and localities.

*Chaetodonaureofasciatus* (Chaetodontidae) from Australia (Kabata 1991). *Chaetodonauripes* Jordan & Snyder (Chaetodontidae) from Seto, Wakayama Prefecture, Japan (Izawa 2016).

####### Current host.

*Chaetodonauriga* and *Chaetodonlunula* (Chaetodontidae).

####### Site of infection.

Gills.

####### Prevalence and mean intensity.

23.1 and 7.3 ± 3.1 (n = 13) to *Chaetodonauriga*; 14.3 and 5 ± 1.4 (n = 14) to *Chaetodonlunula*.

####### Specimens deposited.

CHCM No. 577 (voucher) (1 vial, 1 specimen ♀) (from *Chaetodonauriga*). CHCM No. 578 (voucher) (1 vial, 1 specimen ♀) (from *Chaetodonlunula*). USNM No. 1550606 (voucher) (1 vial, 1 specimen ♀) (from *Chaetodonlunula*).

####### Remarks.

Our samples corresponded to a single mature female from each host, which were not dissected for morphological analysis. Nonetheless, these parasitic copepods resemble *H.bicaudata* in its habitus, antenna, maxilla, and armature of legs 1 and 2, as well as in its preferred hosts, which are butterfly fishes distributed in warm waters from Australia to Japan (see Izawa 2016).

#### Kroyeriidae Kabata, 1979

##### *Kroyeria* van Beneden, 1853

###### Kroyeria longicauda

Taxon classificationAnimaliaSiphonostomatoidaKroyeriidae

Cressey, 1970

####### Type host.

*Carcharhinuslimbatus* (Müller & Henle) (Carcharhinidae).

####### Other host and localities.

*Carcharhinuslimbatus* (Carcharhinidae) from Florida. *Carcharhinusbrevipinna* (Müller & Henle) (Carcharhinidae) from Madagascar (Deets 1994).

####### Current host.

*Carcharhinusmelanopterus* (Carcharhinidae).

####### Site of infection.

Gills.

####### Prevalence and mean intensity.

40 and 16 ± 2.8 (n = 5).

####### Specimens deposited.

CHCM No. 579 (voucher) (1 vial, 1 specimen ♀). USNM No. 155607 (voucher) (1 vial, 1 specimen ♀).

####### Remarks.

The family Kroyeriidae comprises three genera, *Kroeyerina* Wilson, 1932 with nine species, *Kroyeria* with 15 species, and *Prokroyeria* Deets, 1987 with a single species (Walter and Boxshall 2018). Within *Kroyeria*, *K.longicauda* can be identified by the lateral tine on the deeply incised, bifid dorsal stylet, the lateral cuticular flange on the caudal rami, and the small number of unusually large endopodal denticulations of legs 1 to 4 that are unique to this species (Deets 1994).

#### Lernanthropidae Kabata, 1979

##### *Lernanthropus* de Blainville, 1822

###### Lernanthropus

Taxon classificationAnimaliaSiphonostomatoidaLernanthropidae

sp.

####### Current host.

*Kyphosuscinerascens* (Forsskål) (Kyphosidae).

####### Site of infection.

Gills.

####### Prevalence and mean intensity.

50 and 2 (n = 2).

####### Specimens deposited.

CHCM No. 580 (voucher) (1 vial, 1 specimen ♀).

####### Remarks.

The genus *Lernanthropus* includes about 120 species and it is one of the commonest genera of parasitic copepods on marine fishes. In this study, a single female of *Lernanthropus* sp. was collected. We were unable to proceed with the species identification because of the lack of specimens for dissection, which is necessary to observe appendages of the cephalothorax as well as legs 1 and 2. Even with enough material, the identification of *Lernanthropus* sp. is quite difficult because many species have not been described with sufficient detail (Koyuncu et al. 2012).

## Discussion

The present study is the first detailed survey of the diversity and ecological attributes of the parasitic copepods infecting fishes at Palmyra Atoll. All records we report here are new geographical records. Most copepods (10 of 17) belonged to the family Caligidae. Of these ten caligid species, six were in the genus *Caligus* and two in the genus *Lepeophtheirus*. These finding are in agreement with the fact that *Caligus* copepods are mostly found on warm water fishes, while *Lepeophtheirus* copepod diversity is low in the tropics (Ho and Lin 2004, Suárez-Morales and Gasca 2012, Morales-Serna et al. 2016). However, as far as we know, specific evolutionary or ecological mechanisms underlying this greater diversification *Caligus* species in the tropics are not well understood. On the other hand, in experiments carried out by Bravo et al. 2010, they suggest that species of *Caligus* are more active swimmers than species of *Lepeophtheirus*, which in turns increase transmission between hosts. Clearly, such swimming ability could be contributing to dispersal of *Caligus* and host switching. Several copepods species can parasitize multiple fish species (Dojiri and Ho 2013). This is the case of *C.mutabilis* found on *Lutjanusmonostigma* and *L.fulvus* in the present study however, this species has been reported in at least 13 families of marine fishes from the Atlantic and Pacific oceans (Morales-Serna et al. 2015).

Consistent with observations of the monogenean fauna of Palmyra Atoll fishes (Vidal-Martínez et al. 2017), parasitic copepod richness at Palmyra Atoll qualitatively appears low relative to other localities in the Indo-Pacific region. Most of the fish species we examined (30 of 44) were not parasitized by copepods, even with large sample sizes for some fish species (e.g. *Acanthurustriostegus*, n = 50). Several fishes that were unparasitized at Palmyra have copepod records at other sites. For example, *Acanthurustriostegus, Gymnothoraxpictus*, *Epinephelusmerra* and *Sphyraenabarracuda* have been reported as hosts of at least one species of parasitic copepod in other localities of the Indo-Pacific (Boxshall and Huys 2007, Palm and Bray 2014). Because ectoparasite species richness, host size and age are positively related (Rhode 1993, Muñoz and Cribb 2005), the lack of copepods in some host species could be due to our sampling of only young (*Chanoschanos*) or small individuals (*Sphyraenabarracuda*). Furthermore, the intertidal habitat sampled at Palmyra differs from the more often sampled fore-reef and reef flat habitats, making a direct comparison among studies difficult. More generally, Palmyra’s remoteness may contribute to its depauperate copepod parasite fauna. The Line Islands are far from the Austro-Malayan-Philippine region, the presumed center of origin of Indo-West Pacific (IWP) fishes and their parasites. Because we found fewer copepod species than described from Hawaii, which is still further from the presumed center of origin, we suggest that the remote location of the Line Islands and the particularly small size of Palmyra Atoll also contribute to the depauperate nature of the parasitic copepod fauna.

## Supplementary Material

XML Treatment for Orbitacolax williamsi

XML Treatment for Anuretes serratus

XML Treatment for Caligus confusus

XML Treatment for Caligus kapuhili

XML Treatment for Caligus laticaudus

XML Treatment for Caligus aff. mutabilis

XML Treatment for Caligus randalli

XML Treatment for Caligus

XML Treatment for Caritus serratus

XML Treatment for Lepeophtheirus lewisi

XML Treatment for Lepeophtheirus uluus

XML Treatment for Dissonus similis

XML Treatment for Nemesis

XML Treatment for Hatschekia longiabdominalis

XML Treatment for Hatschekia bicaudata

XML Treatment for Kroyeria longicauda

XML Treatment for Lernanthropus
